# Malaria prevalence among patients with acute undifferentiated fever in secondary hospitals in India

**DOI:** 10.1186/1475-2875-13-S1-P40

**Published:** 2014-09-22

**Authors:** Christel Gill Haanshuus, Rosario Vivek, Deepika Xena, Sara Chandy, Dilip Mathai, Anand Manoharan, Nina Langeland, Bjørn Blomberg, Kristine Mørch

**Affiliations:** 1 National Center for Tropical Infectious Diseases, Haukeland University Hospital, Bergen, Norway; 2 Department of Medicine Unit-1 and Infectious Diseases, Infectious Diseases Training and Research Center, Christian Medical College, Vellore, India; 3 University of Bergen, Bergen, Norway

## Background

The World Health Organization reported approximately one million malaria cases and 500 deaths in India in 2012 based on national surveillance data. Estimated number of deaths range from 20,000 to 200,000. The aim of this study was to identify the malaria prevalence among patients with acute undifferentiated fever in rural hospitals in India.

## Materials and methods

During April 2011-November 2012, 1564 patients aged >5 years were included in a larger fever study from seven secondary hospitals in India located in Assam (Tezpur), Bihar (Raxaul), Chhattisgarh (Mungeli), Maharashtra (Ratnagiri), Andhra Pradesh (Anantapur) and Tamil Nadu (Oddanchatram and Ambur). Routine microscopy, genus-specific mitochondrial PCR and the rapid immunochromatographic test (RDT) Parahit Total™ (Span Diagnostics Ltd, Surat, India) were performed. Species-specific 18S PCR or sequencing was performed on genus PCR positive samples. Samples with discordancy between PCR and RDT were retested by PCR from the extraction step. PCR was considered as gold standard.

## Results

EDTA blood for PCR was available from 1416 patients and 19% were malaria PCR positive. The prevalence ranged from lowest 6% in Oddanchatram (South India) to highest 35% in Ratnagiri (West India). *P. falciparum* single infection was detected in 46%, 38% had *P. vivax*, and 11% mixed infections with *P. falciparum* and *P. vivax*. *P. malariae* was detected in 5%. Compared with PCR, the sensitivity of the RDT was 24% and specificity 99%. The sensitivity of microscopy was 29% and specificity 98%. A trend of incorrectly diagnosing *P. falciparum* as *P. vivax* by microscopy was found.

## Conclusions

Our findings support that malaria is an important differential diagnosis in acute undifferentiated fever in all parts of India, and PCR identified a high level of submicroscopic malaria. The very low sensitivity of the RDT emphasizes the importance of choosing a test with high sensitivity, for which WHO’s validation of RDTs should be used.

